# Neurodegeneration as a dysregulation of neuroimmune crosstalk

**DOI:** 10.1016/j.cell.2026.07.015

**Published:** 2026-08-20

**Authors:** F. Chris Bennett, Soyon Hong, Ning Jiang, Ivan Marazzi

**Affiliations:** 1 Department of Psychiatry, Perelman School of Medicine, University of Pennsylvania, Philadelphia, PA, USA; 2 Division of Neurology, Department of Pediatrics, Children’s Hospital of Philadelphia, Philadelphia, PA, USA; 3 UK Dementia Research Institute, Institute of Neurology, University College London, London, UK; 4 Department of Bioengineering, University of Pennsylvania, Philadelphia, PA, USA; 5 Institute for Immunology and Immune Health, University of Pennsylvania, Philadelphia, PA, USA; 6 Center for Cellular Immunotherapies, University of Pennsylvania, Philadelphia, PA, USA; 7 Abramsom Cancer Center, University of Pennsylvania, Philadelphia, PA, USA; 8 Institute for RNA Innovation, University of Pennsylvania, Philadelphia, PA, USA; 9 Center for Precision Engineering for Health, University of Pennsylvania, Philadelphia, PA, USA; 10 Department of Biological Chemistry, University of California, Irvine, Irvine, CA, USA; 11 Center for Epigenetics and Metabolism, University of California, Irvine, Irvine, CA, USA

## Abstract

Neurodegeneration is increasingly recognized not only as a disorder of neurons but also as a breakdown of dialogue between the nervous and immune systems. Recent discoveries reveal that immune cells and inflammatory signals are deeply interwoven with brain function across the lifespan. Far from passive responders, immune cells act as sentinels and shapers of neuronal resilience, vulnerability, and repair. Together, robust data support a model in which neurodegeneration emerges from complex interactions between neural and immune networks, positioning the immune system as both a sensor and driver of brain health. This perspective synthesizes a growing body of work arguing that neurodegenerative diseases are a failure of neuroimmune crosstalk—where protective signals are lost, and maladaptive responses take hold. By restoring immune homeostasis, fine-tuning inflammatory responses, or targeting epigenetic regulators of the immune state, it may be possible not only to slow degeneration but also to promote recovery. We outline the key challenges and opportunities for this paradigm shift and highlight how a deeper integration of neuroscience and immunology could transform the future of treating neurodegenerative diseases. Lastly, we describe critical focus areas to improve our understanding of neurodegeneration and highlight the development of immune-based therapeutics for neurodegeneration.

## INTRODUCTION

The classical view of the central nervous system (CNS) as immune-privileged has been substantially revised. The brain is in continuous dialogue with the immune system across the lifespan. CNS borders, including the meninges, choroid plexus, and associated vascular and lymphatic structures, function not as impermeable barriers but as immunological interfaces,^1^ where diverse effector populations interact with brain-resident cells to monitor and respond to the needs of neural tissue.^2^ Among these, lymphocytes, including CD4^+^ and CD8^+^ T cells, were long thought to be excluded from brain biology but are now recognized to access border compartments and, under defined conditions, the parenchyma itself. Their consequences are context-dependent: in some settings, lymphocytes constrain pathology, while in others, they exacerbate tissue injury in disorders including Alzheimer’s disease (AD)^3,4^ and Parkinson’s disease (PD).^5,6^

These observations are part of a broader body of data supporting a model in which neurodegeneration cannot be explained solely as a cell-autonomous failure of neurons or as a process confined to the CNS. Recent work argues instead that neurodegenerative diseases emerge from dysregulation of a neuroimmune ecosystem spanning brain-resident cells, border tissues, and peripheral immune compartments. Although clear in concept, our understanding of this dialogue remains fragmentary, particularly in humans, and the specificity, timing, and directionality of the relevant signals are only beginning to be defined. In this perspective, we argue that dysregulated neuroimmune communication is not a downstream consequence of neurodegeneration but a pervasive contributor to disease pathogenesis, i.e., a ‘‘concause’’ of neurodegeneration: not necessarily the sole initiating event, but a concurrent and mechanistically meaningful contributor that interacts with neuronal and glial vulnerability to influence disease onset and progression.

Rather than comprehensively reviewing all immune components or disease states, we substantiate this view by first elaborating on the previously underappreciated magnitude of CNS-immune communication (The widening landscape of CNS-immune communication circuits). Then we organize recent advances into three emerging frameworks for how neuroimmune dysfunction may shape disease: the “*outside-in*” framework (Outside-in: Peripheral adaptive immunity as a driver of neurodegeneration), where adaptive immune responses initiated beyond the CNS target or reshape neural tissue; the “*inside-out*” framework (Inside-out: Microglia as orchestrators of neuroimmune communication), where CNS-resident myeloid cells orchestrate broader immune communication; and the “*locked-in*” framework (Locked-in: Gene regulatory dependencies of neuroimmune states), where gene regulatory mechanisms stabilize maladaptive neuroimmune states over time. We then consider how this conceptual shift may guide therapeutic development and future research (From framework to therapy).

## THE WIDENING LANDSCAPE OF CNS-IMMUNE COMMUNICATION CIRCUITS

Emerging data collectively suggest that dialogue across immune-brain circuits shapes neuronal function throughout life, engaging nearly all immune and neural cell types in a continuous dialogue. Microglia and border-associated macrophages (BAMs) serve as innate sentinels,^7–9^ providing local surveillance and rapid effector responses, whereas lymphocytes, once thought excluded from the CNS, confer antigen specificity, immunological memory, and the capacity for long-range coordination.^10^ These arms do not operate independently but as interdependent components of a single system that supports neural integrity in health and can promote pathology when dysregulated.^11^ CD4^+^ T cells, for example, influence microglial maturation and synaptic refinement during development^12^ and continue to shape tissue integrity, neurogenesis, and inflammatory tone in adulthood.^13,14^ Other studies implicate diverse additional immune cell types, including natural killer (NK) cells,^15^ mast cells,^16^ neutrophils,^17^ γδ T cells,^18^ and B cells,^19^ in brain development, health, and disease. Even cells at the interface between adaptive and innate immunity participate. For example, group 2 innate lymphoid cells, involved in tissue repair and homeostasis, influence brain wiring during development in mice.^20^ Neuroimmune communication is thus not only a response to perturbation but also an ongoing homeostatic process woven into normal CNS function, engaging the full spectrum of immune cell types.

Much of this dialogue is organized at CNS border compartments, the blood-brain barrier, choroid plexus, meningeal lymphatics, skull marrow, and circumventricular organ,^1,21–24^ each with distinct cellular composition and regulation. The meninges, in particular, have been extensively characterized as organized immune interfaces enriched in lymphoid (e.g., B and T cells) and myeloid (e.g., macrophages and dendritic cells) populations. These compartments are not static: antigen-experienced, blood-borne B cells accumulate at CNS borders with aging, consistent with an immune composition that is both compartmentalized and responsive to physiological state. Innate lymphoid cells occupy dural sinuses under homeostatic conditions and respond to injury. The choroid plexus hosts lymphocyte subsets at baseline^25^ but transforms into an immune-active niche that regulates trafficking and signaling during neuroinflammation,^21,26^ and direct communication with the meninges allows the skull marrow to provide specialized CNS myeloid cells independent of the circulation. These borders also support active immune regulation: presentation of CNS-derived self-peptides at brain borders can tolerize T cells and limit autoimmunity,^27^ and meningeal regulatory T cells modulate pain signaling in mice.^28^ Recent work in mice further demonstrates that these niches are responsive not only to systemic state but also to neuronal activity itself: neuronal hyperactivity along the perforant pathway recruits antibody-secreting B-lineage cells, likely from meningeal niches, into the adult hippocampus, where locally produced immunoglobulin M (IgM) is required for C1q deposition and selective elimination of presynaptic terminals, suggesting an innate-adaptive crosstalk mechanism for activity-dependent synapse remodeling in the non-diseased adult brain.^29^ CNS borders thus provide complex partitioning of immune cells, supporting nuanced, context-dependent neuroimmune dialogue rather than immune exclusion.

Neuroimmune communication, however, extends well beyond CNS borders to include nearly all peripheral organs and tissues. The gut represents perhaps the best-characterized example: T cells primed in gut-associated lymphoid tissue can traffic to the brain and preferentially localize to disease-relevant regions,^6^ gut-derived IgA-secreting plasma cells protect the meningeal venous sinuses,^30^ and gut microbiota composition shapes microglial states and CNS-immune homeostasis.^22^ More broadly, peripheral immune education, shaped by the microbiome, systemic inflammation, and circulating factors, can alter the composition and functional properties of immune cells that subsequently access CNS borders and parenchymal compartments. Peripheral signals also reach the brain through non-cellular routes: the vagus nerve senses and relays peripheral immune information to central circuits,^31^ neural reward pathways can modulate peripheral immune responses,^32^ and circulating immune molecules, including exercise-induced factors, can directly influence neuroinflammation.^33^ Together, these observations argue against a binary view of “inside” versus “outside” immunity in the CNS and instead support a model in which immune surveillance is spatially distributed across the body, dynamically regulated, and integrated with neural function.

Although operative throughout life, the consequences of immune-CNS dialogue become especially pronounced in aging^34^ and in age-associated neurodegenerative disorders. As one frame-shifting example, T cell infiltration and activity are now robustly implicated in AD,^35–37^ PD,^5^ dementia with Lewy bodies (DLBs),^38^ and amyotrophic lateral sclerosis (ALS).^39,40^ Importantly, the effects of immune activity are context dependent. In mouse models of tauopathy and white matter degeneration, microglia-dependent recruitment of CD8^+^ T cells has been attributed to neurodegeneration.^4,41^ In amyloid mouse models, adaptive immune cells can support protective functions and limit pathology.^42,43^ These apparently contrasting outcomes underscore a central point: neuroimmune interactions are not intrinsically harmful but are shaped by molecular context, disease stage, tissue state, and the specific cell populations engaged.

### Rethinking neurodegeneration—Take-home message 1

**Figure 1.**
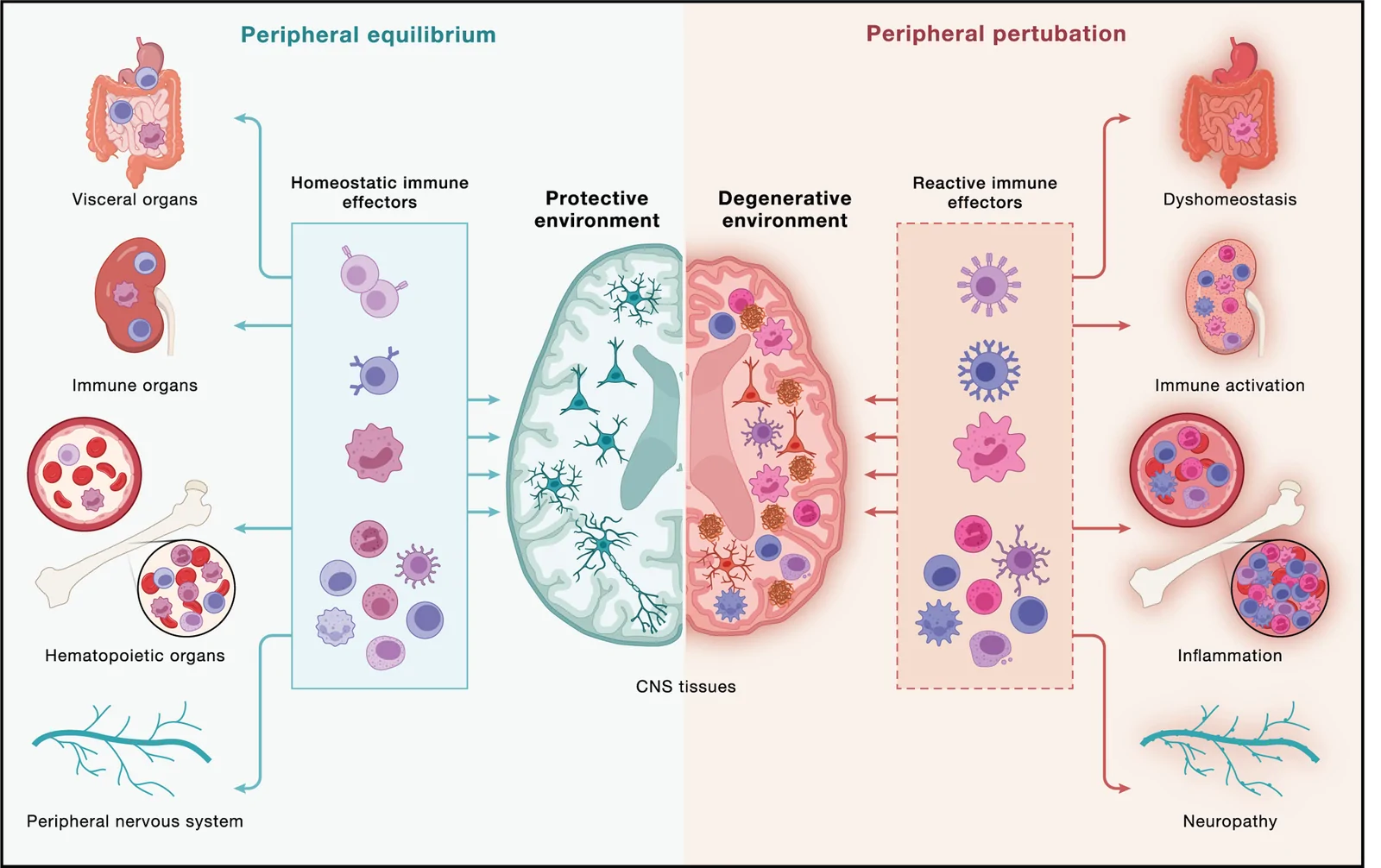
Neuroimmune communication circuits across health and neurodegeneration Immune cells communicate across a continuous, bidirectional circuit between the CNS and periphery. Immune cells from across the body engage CNS tissues and, in turn, are shaped by signals returning from the brain. (Left) Under peripheral equilibrium, immune effectors mobilized from visceral organs, secondary lymphoid organs, hematopoietic tissues, and the peripheral nervous system (PNS)—including CD4^+^ and CD8^+^ T cells, B cells, monocytes (top 3 cell types), NK cells, dendritic cells, mast cells, neutrophils, and innate lymphoid cells (bottom cluster of cells)—engage the CNS in a homeostatic dialogue that supports neural cell health, plasticity, and repair, sustaining a protective environment. (Right) Age-associated dysfunction across the same peripheral compartments reshapes this circuit: altered immune effectors enter the CNS and contribute to shifting the local environment toward a degenerative state in which microglia adopt disease-associated programs, astrocytes become reactive, and neurons accumulate pathology. This model underscores how the same circuitry can yield protective or pathogenic outcomes and that neurodegeneration is downstream of dysfunctional brain-immune crosstalk.

Taken together, these observations support a conceptual reframing. Immune cells, including those outside the CNS, are integral to brain function, and the distinction between “immune” and “neural” signals is not as clear-cut as once assumed. Many molecules classically assigned to one system are produced and sensed by cells of both, linking CNS and peripheral immunity through bidirectional exchange. Prime examples of this are cytokines,^44,45^ complement,^29,46,47^ and MHC-I,^48^ which were classically assigned to immunity but have been shown to be produced by, or sensed by, CNS cells and perform core circuit functions, including developmental synapse elimination and circuit wiring. Neuroimmunity is not an interface between separate systems but an integrated dialogue whose dysfunction can contribute to neurodegenerative decline (Figure 1).

Although the mechanisms linking dysregulated neuroimmune dialogue to pathogenesis remain incompletely resolved, several recurring patterns are emerging. The sections that follow elaborate three: outside-in signals, in which immune responses initiated outside the brain shape, and in some cases perturb, neural tissue; inside-out signals, in which CNS-resident microglia orchestrate broader immune communication; and a locked-in framework, in which gene regulatory mechanisms stabilize neuroimmune states, encoding persistent programs that may constrain subsequent responses.

## OUTSIDE-IN: PERIPHERAL IMMUNITY AS A DRIVER OF NEURODEGENERATION

One dimension of the neuroimmune dialogue arises from peripheral immune activity, where adaptive immune responses initiated outside the CNS can reshape local immune states or directly engage brain-resident cells. A defining feature of adaptive immunity is antigen-specific recognition. T cells recognize peptides presented on major histocompatibility complex (MHC) molecules, termed pMHC, by antigen-presenting cells (APCs) in lymphoid organs, with MHC-I generally engaging CD8^+^ cytotoxic T cells and MHC-II engaging CD4^+^ helper T cells.^49–54^ Together with co-stimulatory signals on APCs, T cells can be activated and differentiate into effector or regulatory states that traffic to tissues: CD8^+^ T cells can directly eliminate target cells, CD4^+^ T cells coordinate immune responses, and regulatory T cells restrain self-reactive responses.^55^ Mechanistic studies support context-dependent protective and detrimental contributions of T cells to neurodegenerative disease, as elaborated below. The central questions are whether disease-associated self-antigens or aggregation-associated neoantigens—arising from altered or misprocessed proteins—are sufficient to engage these adaptive circuits in ways that influence CNS pathology and where such engagement initially occurs.

### T cell responses to neuronal self-antigens

Emerging data suggest that adaptive immunity can mount responses against neuronal self-antigens and that these responses can arise outside the CNS. In PD, peripheral T cells from patients recognize α-synuclein-derived peptides presented on both MHC-I and MHC-II,^5^ indicating that protein aggregation is associated with antigen-specific immune activation in humans.

In juvenile ALS (ALS4), oligoclonal CD8^+^ T cell populations are detected early in patients, in both blood and brain, and expand alongside disease progression, consistent with antigen-driven responses. In mouse models, these cells contribute to pathology, whereas bone marrow replacement, i.e., exchange of the peripheral immune system with healthy donor cells, mitigates neurodegeneration.^39^ Further, TDP-43 mislocalization, a hallmark of ALS in which the normally nuclear RNA-binding protein accumulates in the cytoplasm, can generate cryptic epitopes, i.e., novel peptide sequences not normally exposed, that activate CD8^+^ T cells, including in patient-derived samples.^40^ In a subset of ALS patients carrying pathogenic repeat expansions of *C9orf72*, T cell reactivity to C9orf72-derived peptides has also been reported.^56^

The strength of these observations lies in their convergence across human studies and experimental models, although mechanistic resolution and causal interpretation remain incompletely defined across systems. Collectively, they suggest that neuronal dysfunction can generate antigens capable of engaging adaptive immunity, challenging the view that such responses are solely secondary to tissue damage.

### The gut-brain neuroimmune axis

As introduced in The widening landscape of CNS-immune communication circuits, peripheral tissues, particularly the gut, represent a major site of immune education with potential consequences for brain homeostasis. Beyond microbiota-driven effects on microglia and CNS-immune tone,^57,58^ peripheral immune states can shape the composition and functional properties of circulating lymphocytes, which may subsequently access CNS borders^59^ or parenchymal compartments.^6^ Consistent with this, gut-educated plasma cells (antibody-producing cells) and T cells migrate to CNS borders and influence homeostasis. As noted above, gut-derived IgA-secreting plasma cells defend the meningeal venous sinuses,^30^ and gut-educated T cells traffic to the subfornical organ to regulate behavior in mice.^22^ Intestinal inflammation and dysbiosis can further expand microbiota-responsive T cell populations that migrate to the CNS and alter neuroinflammatory states in animal models.^59–61^

In synucleinopathies, this framework extends to disease-relevant and potentially antigen-specific responses. In mouse models, T cells primed in the gut by macrophages that have engulfed α-synuclein aggregates, the pathological hallmark of PD, traffic to the brain and localize to disease-relevant regions.^6^ Disruption of this circuit, through depletion of gut macrophages or T cells, reduces α-synuclein propagation along the gut-brain axis and ameliorates neurodegeneration. These findings provide functional support, in animal models, for a link between peripheral immune education and CNS vulnerability. In patients, α-synuclein aggregates are detected in peripheral tissues, including the gut, in some cases before overt CNS disease is clinically apparent.^62^ Prodromal features such as constipation coincide with α-synuclein pathology in the enteric nervous system (ENS),^63,64^ where tissue-resident macrophages have been shown in mice to recruit and activate T cells.^6^ These observations raise the possibility that peripheral antigen exposure may generate antigen-specific T cells^5^ that later access the CNS and influence disease-relevant circuits. Within the brain, microglia and BAMs may further shape such responses by adopting antigen-presenting or pro-inflammatory states.^65^ Direct evidence for a complete peripheral-to-CNS circuit in humans remains limited, and the relative contributions of peripheral versus CNS-local priming likely differ across disease contexts. Similarly, the site of priming for recently identified cryptic peptide-specific T cells in TDP-43 proteinopathies remains unclear: these cells could arise within the CNS or in peripheral tissues where disease-relevant antigens are present.

Together, these observations position peripheral immune dysregulation, including within the gut, as a plausible contributor to neurodegeneration, challenging a strictly CNS-centric view of neurodegeneration, and suggest that peripheral tissues may offer both early biomarkers and opportunities for therapeutic intervention.

### Rethinking neurodegeneration—Take-home message 2

Maladaptive immune responses, shaped by self-antigens or aggregation-associated neoantigens, influenced by peripheral immune education, and, in some settings, sustained within the CNS, may amplify and, in selected contexts, potentially contribute to the initiation of disease processes. Their impact is likely to vary across diseases and disease stages, underscoring the need for longitudinal and mechanistically resolved studies in both animal models and human cohorts.

## INSIDE-OUT: MICROGLIA AS ORCHESTRATORS OF NEUROIMMUNE COMMUNICATION

### Microglia in neurodegeneration

Microglia, the long-lived tissue-resident macrophages of the CNS,^66^ are implicated as critical modulators of neurodegeneration through both human genetics and animal models. Impaired handling of protein aggregates,^67^ maladaptive lysosomal function,^68^ aberrant cytokine secretion,^69^ excessive synapse elimination,^47^ and disturbed lipid metabolism^70^ exemplify pathways through which microglia contribute to AD and other disorders, where evidence is drawn from human genetic studies, patient tissue analyses, and mouse models.

**Figure 2.**
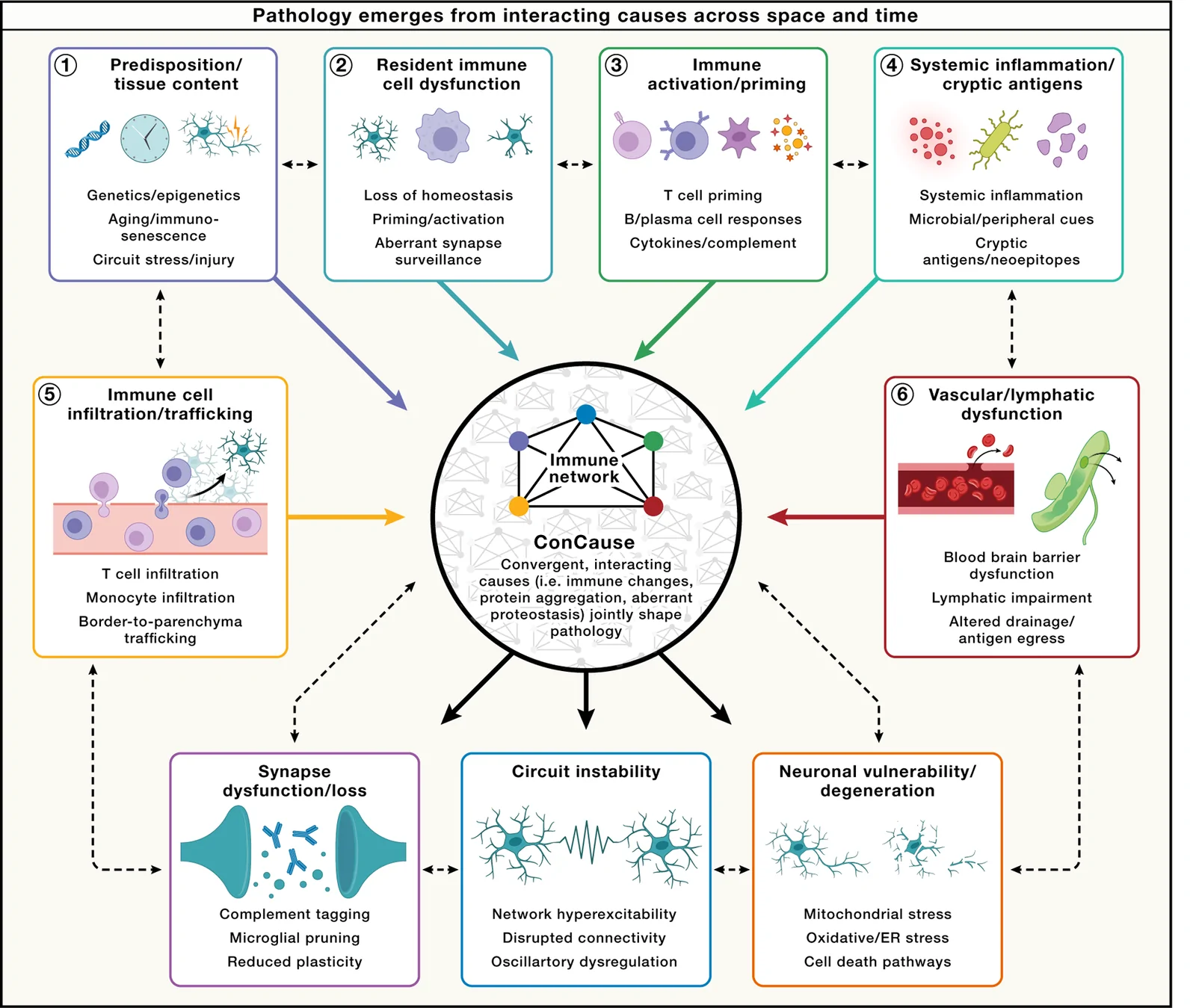
ConCause framework for convergent neuroimmune pathology Multiple interacting processes, including predisposition/tissue context, resident immune cell dysfunction, immune activation/priming, immune cell infiltration/trafficking, vascular-lymphatic dysfunction, and systemic inflammation/cryptic antigens, converge to shape neurodegenerative pathology. These interacting causes, alongside classic contributors like protein aggregation, promote synapse loss, circuit instability, and neuronal vulnerability, while also engaging reciprocal feedback and amplification across space and time. Solid arrows indicate major convergent influences. Dashed arrows indicate feedback and context dependence.

Microglial identity and function are not hardwired but shaped by continuous dialogue with the brain environment.^71^ Although precise mechanisms remain incompletely defined, microglial states, metabolism, and function change with aging in both mice and humans,^72–74^ raising the possibility that aged microglia contribute to vulnerability through impaired homeostatic surveillance, altered inflammatory responses, or inadequate repair. Microglial contributions to disease can therefore be understood within a gene-environment framework, and, as conceptualized by Okabe and Medzhitov,^75^ whether microglia protect or harm depends on their capacity to meet the functional demands of the aging or diseased brain. In this sense, microglia are not passive bystanders but one of the immunologic concauses of disease, capable of nucleating amyloid pathology in mice,^67^ driving synapse loss,^47^ and amplifying neuroinflammation^76^ (Figure 2).

### Microglia as signaling hubs

Microglia-intrinsic dysfunction, particularly within the endolysosomal system (intracellular degradation and recycling pathways), has emerged as a potential concause of neurodegeneration. In mouse models, progranulin loss-of-function results in lysosomal dysfunction and microglial impairment,^77^ and diverse lysosomal insults can reshape myeloid states in ways relevant to disease.^78^ How these altered states influence communication with the broader immune system, however, remains incompletely understood.

This gap is significant because microglia act as central integrators within the CNS, engaging not only with neurons^9,29,79^ and glial partners such as astrocytes^80,81^ and oligodendrocytes^82^ but also with border-associated populations, including in the vasculature^8,83–85^ and meninges.^86^ These interactions have functional consequences for brain homeostasis, including through regulation of CSF flow dynamics,^87^ which holds potential to influence export of neuronal antigens and synapse remodeling. Microglia therefore operate not simply as phagocytes but as signaling hubs that couple local tissue states to broader immune responses.

### Aging, microglia, and T cell recruitment

This hub function becomes particularly relevant in the context of aging, which is the strongest known risk factor for many neurodegenerative diseases.^88^ The aging brain is characterized by blood-brain barrier dysfunction, observed in both human neuroimaging studies^89^ and mouse models,^90^ which contributes to neural dysfunction.^91^ Age-related changes in circulating factors and endothelial cells further shape the immune milieu in ways relevant to neuroinflammation.^92,93^

With age, microglia adopt reactive states characterized by altered surveillance and maladaptive responses in mice,^94–97^ including secretion of chemokines such as CXCL10 that recruit CD8^+^ T cells to the CNS.^41^ Once present, T cells can feed back onto microglia, amplifying inflammatory tone and exacerbating neuronal injury in mouse models. A well-characterized example of this circuit comes from experimental autoimmune encephalomyelitis (EAE), a mouse model of multiple sclerosis, in which microglia present CNS-derived antigens to infiltrating T cells via MHC-II, driving reciprocal activation and shaping T cell responses.^98^ Whether similar antigen presentation occurs in neurodegenerative disease remains an open question. Current evidence is largely associative, although the molecular machinery is present.

In models of AD and tauopathy, microglial responses to pathology are associated with T cell recruitment and functional interaction. Antigen-experienced CD8^+^ T cells are detected in the CSF and parenchyma in AD patients,^37^ and T cells modulate microglial activity and disease progression in mouse models.^4,41,99^ Mechanistically, microglia-derived CXCL10 recruits CD8^+^ T cells that induce interferon-responsive states in microglia and oligodendrocytes, contributing to white matter degeneration in aging mice. In tauopathy models, microglia-mediated T cell infiltration exacerbates neurodegeneration through both direct neuronal injury and amplification of inflammatory signaling.

These interactions are not uniformly detrimental. Amyloid-β (Aβ)-specific regulatory T cells attenuate pathology in mouse models,^100^ and modulation of T cell trafficking or state improves pathology and cognition in preclinical studies.^43,101–103^ Immune checkpoint pathways further regulate this balance: TIM-3, expressed on both T cells and microglia, restrains microglial phagocytic activity and modulates AD pathology in mice,^104^ identifying checkpoint signaling as a mechanism controlling neuroimmune interactions.

### Microglia and the spatial spread of pathology

An additional dimension of microglial involvement is their emerging roles in shaping aggregate pathology and its spatial propagation across brain regions. Microglia do more than detect and clear plaques.^105^ In mouse models of Aβ proteinopathy, microglia contribute to the generation of Aβ pathology^67,106^ and, through TREM2- and APOE-dependent mechanisms, compact plaques to constrain their growth.^107,108^ Microglia have also been implicated in the spread of Aβ pathology beyond individual plaque niches.^109^ Likewise, in mouse models of tauopathy, microglial depletion or inhibition of exosome synthesis suppresses tau propagation from the entorhinal cortex to the hippocampus,^110^ and manipulation of microglial nuclear factor κB (NF-κB) signaling causally alters tau seeding and spread.^111^ Human genetic studies complement these findings: variants associated with microglial activation states show region-specific relationships with neuropathological features across aging and disease,^112,113^ and the association between TREM2 expression and AD neuropathology may itself differ across brain regions.^114^ Together, these observations support the model in which microglia help determine where and how pathological aggregates form, propagate, and exert their effects. Integrating this systems-level perspective with local cell-cell interactions will be important for understanding how neuroimmune processes scale to produce the regional patterns of neurodegeneration observed in patients.

### Beyond microglia: BAMs, astrocytes, and oligodendrocytes

Many of the properties described above likely extend to BAMs, which reside at key CNS-immune interfaces, including perivascular spaces, meninges, and the choroid plexus, where they directly engage circulating immune cells (reviewed in Silvin et al.^115^). Positioned at these entry points, BAMs are well placed to integrate peripheral immune signals with CNS-resident responses and have been implicated in functions such as CSF flow regulation^87^ and Aβ clearance.^116^ Depletion or perturbation of BAMs in AD mouse models can alter pathological features, including aggregate burden and vascular dysfunction, supporting a potential functional role in disease.^117^ In synucleinopathy models, BAMs have also been implicated as APCs that recruit T cells,^65^ suggesting a role in linking peripheral immune activation to CNS responses. More broadly, BAM activation can shape microglial synapse phagocytosis, indicating bidirectional crosstalk between the two populations.^8^

Beyond myeloid cells, astrocytes and oligodendrocytes also contribute to neuroimmune signaling and disease processes. Astrocytes are increasingly recognized as central regulators of immune activity within the CNS, where they influence microglia,^118,119^ crosstalk with T cells,^120,121^ and restrain immune responses through cytokine-mediated reprogramming and checkpoint pathways (e.g., interleukin [IL]-27, PD-L1, and TRAIL).^122^ Through these roles, astrocytes can regulate the magnitude, localization, and duration of T cell responses within the CNS. Consistent with this, astrocyte dysfunction has been implicated in multiple neurodegenerative contexts. For example, in ALS models, TDP-43 mislocalization in astrocytes leads to motor deficits, potentially through disruption of metabolic support to neurons,^123,124^ and may also contribute to cognitive impairment via altered chemokine-mediated astrocyte-neuron interactions.^125^ Astrocyte dysfunction has similarly been linked to tau pathology and to aberrant synaptic regulation,^119^ connecting proteinopathy to the immune and homeostatic functions that astrocytes exert under physiological conditions. These observations together position astrocytes as key modulators; however, how astrocytes and microglia jointly shape immune tone in the CNS is an area of active investigation.

Oligodendrocyte-lineage cells are also emerging as participants in neuroimmune communication. Oligodendrocytes can present antigens via MHC-I to CD8^+^ T cells,^126^ providing a potential route for direct immune-mediated injury, while oligodendrocyte precursor cells (OPCs) can adopt inducible immune-like states under inflammatory conditions.^127^ In line, T cells localize in close proximity to immune-activated oligodendrocytes in white matter,^34^ supporting the existence of interactions within relevant CNS niches. At the same time, oligodendrocyte function is tightly coupled to microglial state: microglia regulate oligodendrocyte differentiation and CNS myelin growth and integrity,^82,128^ highlighting a bidirectional axis between immune activation and myelin repair.

Collectively, these observations position microglia not as isolated effectors but as nodes within a broader, multicellular network in which BAMs relay peripheral signals, astrocytes tune local immune responses, and oligodendrocytes both respond to and participate in immune interactions. A comprehensive treatment of each of these cell types is beyond the scope of this perspective, but this integrative view underscores that neuroimmune communication extends beyond microglia-T cell interactions to a distributed cellular network that shapes disease vulnerability and progression.

### Rethinking neurodegeneration—Take-home message 3

Taken together, these findings position microglia as central regulators of neuroimmune communication in health and disease. Immune networks, exemplified by microglia-T cell interactions, are neither fixed nor intrinsically harmful but dynamic exchanges that shift across development, aging, and disease. While these interactions can become maladaptive in the context of chronic inflammation or proteinopathy, they also retain the capacity to support repair and homeostasis. Deciphering the rules governing this neuroimmune code, i.e., how antigen presentation, tissue state, and checkpoint signaling shape outcomes, will be essential for translating mechanistic insight into therapeutic strategies. Ultimately, addressing neurodegeneration may require not only targeting protein aggregates but also recalibrating immune communication networks to restore equilibrium within the brain.

## LOCKED-IN: GENE REGULATORY DEPENDENCIES OF NEUROIMMUNE STATES

Neurodegenerative diseases have traditionally been framed in genetic terms, in which pathogenic mutations initiate cellular dysfunction and, ultimately, neuronal loss. However, this view does not readily account for the marked variability in disease onset, progression, and severity, nor the influence of lifelong exposures on disease trajectories. Gene regulatory mechanisms, including chromatin-based processes, provide a complementary layer through which environmental and experiential inputs shape gene expression without altering the underlying DNA sequence. While genetics represents a fixed canvas (the genome), epigenetics acts as a dynamic brush through which nature and nurture converge. This convergence paints new landscapes over time through the establishment of time-aware gene regulatory networks (GRNs). In fact, at a fundamental level, whether we are talking about an immune cell, a neuronal cell, or any other cell type, cellular identity, differentiation, and response to external stimuli are controlled GRNs. These systems operate and are established on chromatin. Chromatin is thus a storage of information and acts as a dynamic platform that relays transcriptional responses to internal and external signals. Within the neuroimmune system, this regulation provides a framework through which prior exposures, including inflammatory signals or changes in neuronal activity, can bias subsequent cellular responses, giving rise to persistent changes in cellular function sometimes described as cellular memory. For example, neuronal early-life stress exposure can induce lasting molecular changes associated with altered behavior or stress responsiveness. These processes are best understood as within-lifespan, environmentally responsive adaptations, rather than mechanisms of transgenerational inheritance (Box 1).

Two simple features link gene regulatory mechanisms to neurodegeneration in a manner consistent with a network-based view of disease.

First, many genes implicated in neurodegenerative diseases encode proteins that regulate transcriptional programs or modulate chromatin architecture,^139^ shaping the capacity of cells to mount appropriate gene expression responses. For example, TDP-43, along with FUS (the causative gene in ALS6), are RNA-binding proteins with established roles in transcriptional regulation and RNA splicing, and their mislocalization (TDP-43) or mutation (FUS) is associated with widespread disruption of gene expression.^140^ Similarly, expansions in *C9orf72*, a leading genetic cause of ALS and frontotemporal dementia (FTD), generate toxic RNA foci and abnormal protein products that interfere with RNA metabolism and chromatin remodeling.^141^ In Huntington’s disease (HD), mutant huntingtin (HTT) disrupts key transcriptional regulators such as REST and CBP, further illustrating how disease-associated proteins intersect with gene regulatory machinery.^142^ These observations support the view that aspects of neurodegeneration can be considered as disrupted transcriptional homeostasis. Notably, perturbations in these regulatory systems may alter how cells interpret and respond to environmental and intercellular signals, constraining adaptive responses within the neuroimmune network.

Second, key components of these regulatory systems are shared across neuronal and immune lineages, providing a molecular substrate for coordinated, or jointly dysregulated, responses across the neuroimmune network. Transcriptional regulators implicated in neurodegeneration frequently contribute to immune cell function as well. For example, *TDP-43* and *FUS* have been linked to the regulation of inflammatory gene expression in microglia and peripheral immune cells.^143,144^ *MEF2C*, a transcription factor important for synaptic plasticity, also shapes microglial and other immune cell states and is implicated in microglial homeostatic programs relevant to disease.^145^ *NRF2*, known for regulating antioxidant responses in neurons, modulates inflammatory programs in microglia and astrocytes.^146^ Dysfunctional *NRF2* signaling has also been associated with neurodegenerative and autoimmune disorders.^147^ Similarly, *PU.1* is a central regulator of myeloid cell development, including microglia,^148^ directly linking immune transcriptional programs to neurodegenerative contexts.

Additional examples reinforce this convergence. Senataxin (SETX) is a DNA/RNA helicase and transcriptional regulator vital to immune regulation.^149^ Mutations in *SETX* cause ataxia with oculomotor apraxia type 2 (AOA2) and amyotrophic lateral sclerosis 4 (ALS4), both progressive neurodegeneration.^150^ SETX helps neurons resolve R-loops—RNA:DNA hybrids that form during transcription—and maintains genomic stability.^151^ Accumulation of R-loops due to SETX dysfunction leads to both transcriptional stress and DNA damage that likely contributes to neuronal loss but also to immune deregulation of both adaptive and innate arms.

Although not a direct chromatin regulator, C9orf72 also illustrates how perturbations in intracellular processes such as autophagy and endosomal trafficking can influence inflammatory and transcriptional states across neurons and immune cells. Loss of C9orf72 function leads to impaired clearance of cellular debris and aberrant inflammatory signaling. In microglia and macrophages, *C9ORF72* deficiency results in elevated expression of inflammatory cytokines and hyperactive immune responses.^152^ Further, *C9orf72*-deficient T cells show enhanced activation and skewed cytokine production that contributes to systemic immune imbalance.^153^ These immune phenotypes often precede or parallel neurodegenerative symptoms, suggesting that disruptions in cellular homeostasis extend across compartments of the neuroimmune system and that immune dysfunction is not merely a consequence but, in some contexts, a concause of disease.

### Rethinking neurodegeneration—Take-home message 4

Taken together, these observations suggest that neurodegenerative disease arises, in part, from altered control of gene regulatory networks across interconnected neuronal and immune cell populations, rather than from isolated defects within a single cell type. In this framework, gene regulatory mechanisms do not act as primary drivers in isolation but instead provide a stabilizing layer that can constrain or reinforce neuroimmune states over time, particularly under conditions of repeated or chronic perturbation. Within the neuroimmune network, such mechanisms may influence how microglial and astrocytic states are maintained in specific spatial and circuit contexts, shaping processes including synapse remodeling and inflammatory signaling. In this way, acquired or compensatory gene networks may contribute to the persistence of cell states that have been initiated by earlier perturbations, without necessarily determining their initial induction.

In aging, these processes become increasingly relevant. Epigenetic regulation progressively loses fidelity over time, accompanied by increased variability and drift in gene expression and heightened sensitivity to environmental inputs.^154–156^ Accumulated exposures across the lifespan, including inflammatory signals and changes in neuronal activity, may further bias these trajectories, reducing the capacity of neuroimmune cells to appropriately recalibrate their responses. Mutations in genes at the intersection of these processes convert a static genetic defect into a dynamic one and progressively amplify disruption of gene regulatory networks, ultimately contributing to disease.

## FROM FRAMEWORK TO THERAPY

**Figure 3.**
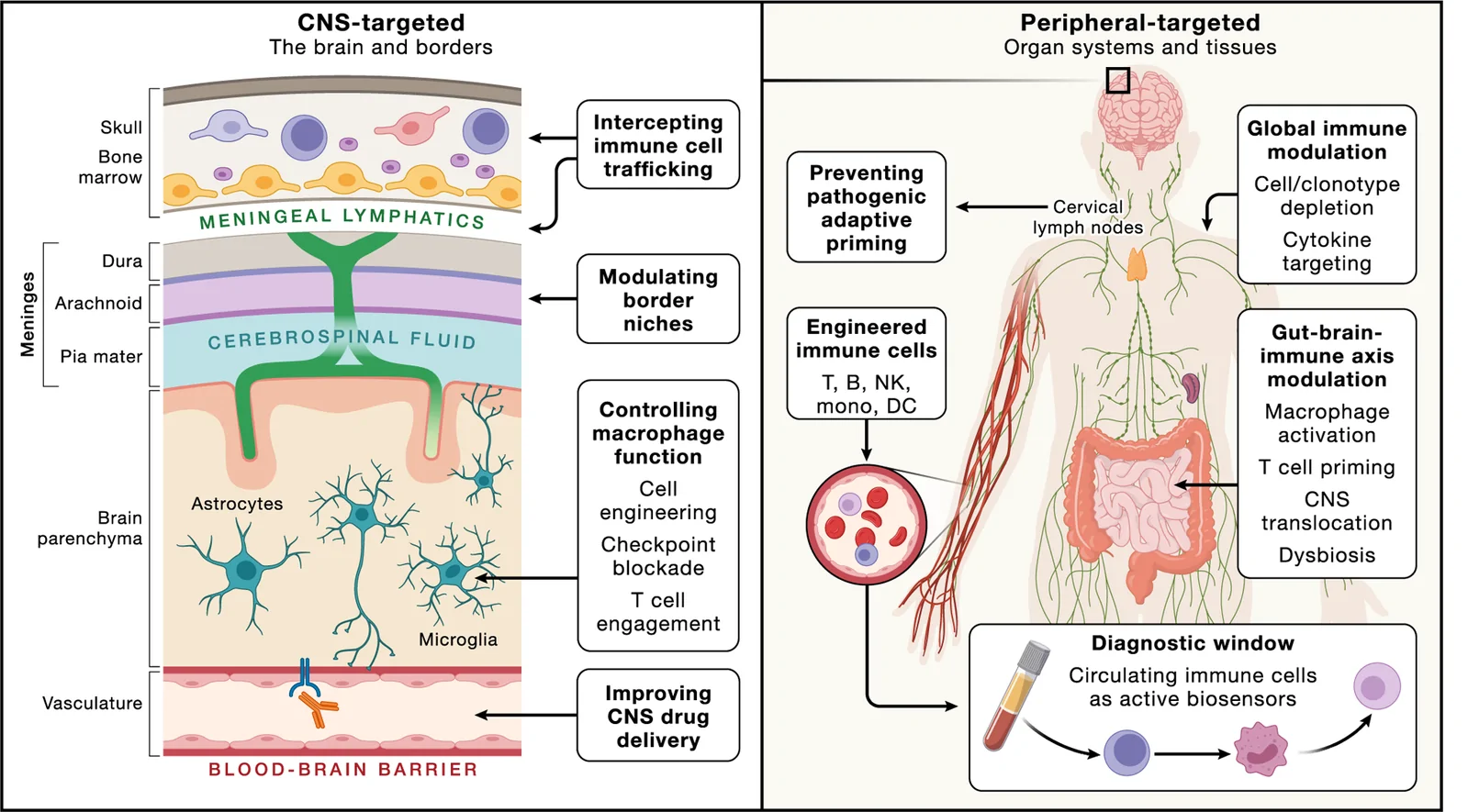
Therapeutic and diagnostic opportunities across the neuroimmune axis in neurodegeneration Neuroimmune circuits offer multiple anatomically distinct points of intervention, both within the CNS and at peripheral sites that instruct CNS immunity. (Left) CNS-targeted strategies act on the brain and its borders: intercepting immune cell trafficking through skull marrow channels and meningeal lymphatics; modulating immune niches at the dura, arachnoid, and pia; controlling parenchymal macrophage function via microglia replacement, cell engineering, checkpoint modulation, and disruption of pathogenic microglia-T cell engagement; and improving delivery of biologics across the blood-brain barrier. (Right) Peripheral-targeted strategies act upstream of CNS pathology: preventing pathogenic adaptive priming in CNS-draining cervical lymph nodes (cLNs), deploying engineered immune cells (T, B, NK, monocytes, and dendritic cells); globally modulating immunity through cell- or clonotype-specific depletion and cytokine-directed biologics; and modulating the gut-brain-immune axis, including peripheral macrophage activation, T cell priming, translocation of misfolded proteins to the CNS, and dysbiosis. Because immune cells recirculate between CNS, border, and peripheral compartments, biosensor-engineered leukocytes may provide a diagnostic window into the brain-immune state, accessible through a routine blood draw and complementary to conventional biomarkers.

The neuroimmune frameworks outlined above highlight therapeutic opportunities in neurodegeneration (Figure 3). Current clinical approaches largely target aggregate pathology itself or the immune cells that engage it (largely microglia). A communication-centered view expands the intervention map to include the upstream circuits that shape immune activity, the tissues in which those circuits are assembled, and the cell-cell signals that mediate them.

### The current landscape

Immunotherapies are already clinically advanced for neurodegenerative disease. Furthest along, lecanemab (Leqembi) and donanemab (Kisunla) are amyloid-directed antibodies approved by the FDA for early AD that act in large part through induction of microglial phagocytosis of amyloid plaques.^157–159^ Additional agents targeting microglia, e.g., TREM2 agonist antibodies, and other aggregate proteins, like anti-α-synuclein antibodies for PD, are at various stages of clinical development.^160,161^ Myeloid replacement therapies are also approaching clinical translation. Hematopoietic cell transplantation (HCT) is used to treat adult-onset leukoencephalopathy with axonal spheroids and pigmented glia (ALSP),^162^ a rare neurodegenerative disorder caused by *CSF1R* mutation, and gene-modified HCT for leukodystrophies, including two FDA-approved products, demonstrates the therapeutic value of engineered myeloid cells.^163,164^ Building on this foundation, engineering donor myeloid cells to enhance TREM2 expression shifts the logic from correcting missing enzymes toward augmenting neuroprotective functions.^165^

These examples demonstrate that immune cells can be tractable therapeutic targets, but these and other emerging strategies largely engage pathologic hallmarks directly or through microglia that engage and help clear them. Advances in drug delivery and target selection will continue to improve them, but the mechanistic opportunities unmasked by recent works argue that greater gains may come from directly modulating the broader immune communication circuits, spanning multiple cell types and tissues, that converge on synapse loss and neuronal death. Three points of emphasis follow from the mechanistic insights discussed in this perspective.

### Targeting immune circuits, from the brain effector back to the peripheral instigator

The fundamental schema of immunity is an arc from tissue change to innate cell reaction to adaptive cell engagement, with each step shaping the next. Within the brain, for example, interferon (IFN)-γ from infiltrating CD8^+^ T cells drives interferon-responsive inflammatory states in microglia that amplify neurodegeneration.^4^ Strategies to disrupt this amplification loop, for instance, by blocking the cytokine signal locally, by modulating its receptor, or by engineering replacement microglia refractory to it, could dampen pathology at the point of cell-cell contact. Just upstream, the mechanisms governing T cell infiltration across CNS borders (endothelial adhesion molecules, meningeal immune niches, and BAM gating functions) offer additional intervention points. Further upstream still, the chemokine signals that recruit T cells, such as the CXCL10-CXCR3 axis through which reactive microglia recruit CD8^+^ T cells that drive white matter degeneration,^41^ could be disrupted to prevent maladaptive recruitment while preserving beneficial immune surveillance. And at the most distal end of this arc, the priming of disease-relevant T cells, whether at brain borders, in cervical lymph nodes draining CNS antigens,^27^ in gut-associated tissue where macrophages present α-synuclein to T cells,^6^ or at sites where cryptic TDP-43 epitopes first engage adaptive immunity,^40^ could be targeted to prevent pathogenic clones from arising in the first place. Immune circuits contributing to neurodegeneration thus contain multiple, potentially additive points of therapeutic access, both close to and far upstream of the pathology they modulate. As our mechanistic understanding of these circuits deepens, so too will information on specific strategies to intercept them, potentially even leading “precision immunology” interventions customized to a patient’s specific pattern of immune circuit dysregulation.

### Expanding the therapeutic map: New tissues and cell types

Intrinsic to a neuroimmune-circuit view is that immune cells distributed across the body are therapeutically relevant at the right stage of the pathological cascade, both near and far from the CNS. Proximal and intuitive targets include the meninges, an organized interface central to CNS-immune cell trafficking; the skull bone marrow, a reservoir of myeloid progenitors that directly supplies the meninges and parenchyma^24^; and cervical lymph nodes, where CNS-derived antigens engage adaptive immune cells.^166^ But more distal targets follow just as directly. Circulating T cells could be engaged to deplete pathogenic clones (e.g., antigen-specific CD8^+^ T cells expanded in ALS4^39^), expand regulatory populations,^167^ or be engineered with modified T cell receptors (TCRs) that deliver regenerative rather than destructive signals.^168^ Gut macrophages, which in synucleinopathy models engulf α-synuclein aggregates and prime T cells that traffic to the brain, could be modulated to break this gut-brain-immune circuit. More broadly, gut microbiome and mucosal immune setpoints could be targeted to suppress the generation of pro-inflammatory lymphocytes that later access the CNS. Fecal microbiota transplantation trials in PD^169,170^ represent early efforts along this axis, although the immunological mechanisms remain to be defined.

Hematopoietic stem and progenitor cells (HSPCs) are another attractive target. Clonal hematopoiesis, the age-associated, often TET2-driven expansion of HSPC clones, leads to altered function of tissue-resident immune cells.^171^ TET2-mutant macrophages infiltrate the CNS, enhance phagocytosis, and mitigate AD progression in mice,^172^ consistent with epidemiological evidence, albeit with conflicting direction of effect, that clonal hematopoiesis mutations modulate AD risk in humans.^173,174^ In another body of work, correcting circulating myeloid cell metabolism can mitigate cognitive decline,^175^ further demonstrating how reshaping the hematopoietic progenitor pool can change the immune cells that ultimately populate the brain.

Further, as the understanding of these circuits deepens, cell types not traditionally linked to neurodegeneration, like dendritic cells that shape neurotoxic T cell priming,^176^ B cells that transit through meningeal borders,^15^ and neurotoxic NK cells that impair cognition,^177^ could emerge as tractable therapeutic targets.

### Modalities: Engineering and communication-modulating biologics

#### Engineered cells as precision effectors

Myeloid replacement offers perhaps the most immediate opportunity for systems-level immunotherapy, given its active clinical use for CNS indications. Preclinical models already deploy engineered donor macrophages with synthetic receptors, such as chimeric antigen receptors (CARs), to enhance phagocytosis of disease-relevant aggregates.^178,179^ Though with different goals, precedents in peripheral diseases suggest this only scratches the surface.^180,181^ It is no longer farfetched to engineer microglial surrogates that modulate T cell behavior (recruitment, local activation) through conditional expression of chemokines or checkpoint ligands or that CAR signaling domains can be tuned to elicit specific macrophage states, for instance, to modulate T cell tolerance upon aggregate encounter.^182^ The conceptual shift is from replacement-as-correction to replacement-as-circuit-rewiring: donor macrophages as engineered nodes that send defined signals to other immune and neural cells.

Engineered T cells represent another frontier, already with some precedent.^183,184^ With antigen targets now identified in PD (α-synuclein^24^) and ALS (cryptic TDP-43 epitopes^78^; C9orf72 peptides^105^), existing CAR-T and TCR-T platforms could in principle be adapted to neurodegeneration: antigen-specific regulatory T cells to suppress pathogenic clones, cytotoxic T cells to eliminate cells harboring specific pathological states, or T cells engineered with signaling that delivers trophic rather than cytotoxic outputs. Translating these concepts will require defining the optimal disease stage for intervention, the neuroimmune context that determines whether engagement is protective or harmful, and the considerable engineering challenges of directing immune cells to the right tissue compartment. Identifying a neurodegeneration-associated antigen is only a first step toward a clinically actionable target.

#### Biologics targeting neuroimmune communication

Biologic platforms offer a parallel strategy. Brain-penetrant delivery technologies, such as transferrin receptor-mediated transcytosis vehicles,^185^ were developed to improve CNS delivery of aggregate-targeting antibodies but could equally deliver biologics that modulate immune communication within the CNS. These may include checkpoint agonists, anti-inflammatory cytokines, or decoy receptors that disrupt chemokine-mediated T cell recruitment at neuroimmune interfaces. In this context, the therapeutic logic for checkpoint modulation in neurodegeneration could be the inverse of oncology: reinforcing immune restraint rather than releasing it. Looking further ahead, engineered biologics designed to modulate specific cell-cell interactions at neuroimmune interfaces, for example, bispecific antibodies that simultaneously engage a microglial receptor and a T cell checkpoint, or engineered cytokine variants with altered receptor selectivity that shift the balance between pro-inflammatory and regulatory signaling at specific tissue sites, represent a natural extension of the communication-targeting logic. The goal is not to deplete or replace cells but to change the conversation between them.

#### Immune cells as diagnostic windows into the brain

A distinctive feature of neuroimmune circuits is that they are not confined to the brain. Immune cells transit between CNS, border, and peripheral compartments, carrying molecular records of the environments they have encountered. This creates opportunities for diagnostics alongside therapeutics. T cell clonality and antigen specificity in blood and CSF already reflect disease-relevant immune activation in ALS4, PD, and AD, and peripheral myeloid cell states could report on the inflammatory programming that shapes CNS-resident immune cells. More ambitiously, because many immune cells naturally recirculate through the CNS, they could in principle be engineered with synthetic reporter circuits and produce detectable signals upon encountering disease-associated antigens, aggregates, or cytokine environments, thereby serving as active biosensors of brain-immune states, accessible through a blood draw. Although still in early development, this approach would complement conventional biomarkers by providing an active rather than passive readout of CNS immunity.

## CONCLUSION: TOWARD A MORE INTEGRATED NEUROIMMUNOLOGY

Neuroimmune communication has emerged not only as a peripheral feature of neurodegeneration but also as a central axis of its pathogenesis. The three frameworks developed in this perspective—*outside-in* adaptive responses that engage the CNS from peripheral tissues, *inside-out* circuits in which brain-resident myeloid cells orchestrate broader communication, and *locked-in* gene regulatory programs that stabilize neuroimmune states—together reframe disease as the dysregulation of a distributed, dynamic network rather than a failure confined to any single cell type or organ. In this setting, immune dysfunction is best understood as a concause of neurodegeneration: a mechanistically meaningful contributor that interacts with neuronal and glial vulnerability to shape disease onset and progression.

The first immunotherapies for neurodegeneration are already in clinical use. The amyloid-directed antibodies lecanemab and donanemab, together with the expanding portfolio of microglia- and aggregate-targeted agents in development, demonstrate that modulation of neuroimmune activity can alter disease trajectories in humans. The frameworks developed here argue that the next generation of therapies will extend beyond targeting pathological aggregates directly, toward modulating the upstream circuits that connect peripheral immune tissues, CNS borders, and brain-resident cells, and in parallel, toward using those circuits as diagnostic windows into the brain.

Realizing this vision will require confronting what remains unknown as well as bold funding initiatives to foster cross-disciplinary, ambitious collaborations. Public funding and visionary private initiatives, such as the Chan Zuckerberg Initiative’s Neurodegeneration Challenge Network, have played an essential role in catalyzing the cross-disciplinary collaboration this work requires. However, we are only at the beginning. The specificity, timing, and directionality of many neuroimmune signals are incompletely defined, as are the conditions under which immune engagement becomes maladaptive rather than protective. Addressing these questions will demand longitudinal, mechanistically resolved studies that track neuroimmune circuits across disease stage and tissue compartment, particularly in humans, and sustained investment that bridges traditional disciplinary boundaries. By embracing this momentum, the community is poised to move from descriptive to mechanistic and functional insight and specific targeted intervention strategies, bringing genuine immunotherapies for AD, PD, ALS, and other currently intractable CNS disorders closer within reach.

## Box 1. Transgenerational inheritance and cellular memory

Epigenetics was initially proposed as a means by which environmental information could be transmitted across generations. Examples in plants and invertebrates (such as small RNA-mediated inheritance in *Arabidopsis thaliana* or *Caenorhabditis elegans*) support this concept,^129,130^ but evidence for stable and functionally relevant transgenerational inheritance in mammals remains limited.^131^ In many cases, phenotypes observed in offspring, such as altered metabolism or stress responses, can be explained by persistent environmental influences or by reprogramming within an individual, rather than by true germline transmission of epigenetic information.

Immune responses are one domain in which transgenerational inheritance is sometimes invoked. There is currently little evidence that the innate immune system communicates via epigenetic memory across generations. However, many studies suggest that infections or immune conditions can influence secondary immune responses. For example, trained immunity, a heightened state of innate immune readiness following infection or vaccination, is thought to reside in hematopoietic stem and progenitor cells (HSPCs). Environmental cues can reprogram HSPCs to produce myeloid cells with altered responsiveness^132^ so that the “memory” resembles an imprint on the bone marrow niche rather than a heritable program passed through generations. This mechanism is thought to confer cells with memory without the need to change DNA, in contrast to adaptive immunity, which uses mutation-dependent antigen-specific memory within T and B cells. Recent work indicates that innate immune memory can also arise through chromatin changes in cells that experience infection, enhancing responsiveness to subsequent infections.^133^

In the nervous system, emerging studies suggest that experiences can leave molecular imprints that persist across generations. Paternal exposure to stress or trauma in mice has been associated with behavioral and neurobiological changes in offspring. For example, male mice exposed to chronic stress tend to have offspring with altered stress hormone responses and anxiety-like behavior.^134,135^ These effects are often linked to changes in sperm’s small RNA expression, reminiscent of metabolic phenotypes inherited in progeny from obese male mice.^136^ In *C. elegans*, neuronal activity can influence the germline and transgenerational gene regulation: in one study, neuronal perception of environmental stimuli led to small RNA-mediated gene silencing that remained for multiple generations,^137^ suggesting a potential route by which sensory experience can shape offspring phenotype.

Despite these findings, definitive evidence of stable transgenerational inheritance or cellular memory in the mammalian nervous system remains limited. Distinguishing inheritance from *in utero* or early-life environmental influence is a challenge, as is demonstrating that enhanced responsiveness arises from the same cell that experienced the first insult. Nevertheless, the abovementioned studies, along with very recent ones,^138^ raise the possibility that immune and neural experiences may leave long-lasting and heritable effects beyond the originally impacted generation, potentially contributing to lineal associations observed across psychiatric, neurological, and immune disorders.
